# Electrochemical and Catalytic Properties of Carbon Dioxide-Activated Graphite Felt

**DOI:** 10.3390/molecules27196298

**Published:** 2022-09-24

**Authors:** Andrzej Świątkowski, Elżbieta Kuśmierek, Ewa Chrześcijańska, Krzysztof Kuśmierek, Andrzej Albiniak

**Affiliations:** 1 Institute of Chemistry, Military University of Technology, ul. gen. S. Kaliskiego 2, 00-908 Warsaw, Poland; 2 Faculty of Chemistry, Institute of General and Ecological Chemistry, Lodz University of Technology, ul. Zeromskiego 116, 90-924 Lodz, Poland; 3 Faculty of Chemistry, Wroclaw University of Technology, ul. Gdanska 7/9, 59-344 Wroclaw, Poland

**Keywords:** graphite felt, activation in CO_2_, voltammetric measurements, catalytic activity, stability

## Abstract

The commercial graphite felt GFA 10 was subjected to an activation process with the use of CO_2_ at 900 °C for 35 and 70 min. Pristine and heat-treated materials were characterized using various methods: low-temperature N_2_ adsorption, SEM, and EDS. Voltammetric measurements of GFA samples (before and after activation) as the working electrode were carried out. Voltammograms were recorded in aqueous solutions of 4-chlorophenol and sodium sulfate as supporting electrolyte. The catalytic activity of GFA samples in the process of 4-chlorophenol oxidation with the use of H_2_O_2_ was also investigated. The influence of graphite felt thermal activation in the CO_2_ atmosphere on its electrochemical and catalytic behavior was analyzed and discussed. Results of the investigation indicate that GFA activated in CO_2_ can be applied as an electrode material or catalytic material in the removal of organic compounds from industrial wastewater. However, the corrosion resistance of GFA, which is decreasing during the activation, needs to be refined.

## 1. Introduction

Among various kinds of carbon materials applied in electrochemistry (as electrode materials in analysis, energy storage, or energy sources), very important are carbon fibers, felt, or cloth. Carbon-based electrodes find many different applications [1]. Electroanalysis can be mentioned here [2,3]. They are also used to remove polluting substances from water [4,5,6]. Another area of use of carbon felt electrodes are batteries [7,8,9,10,11,12,13] and microbial fuel cells [14,15,16]. An important area of research is to characterize [17,18,19,20] and modify [21,22,23,24,25] the properties of carbon felts.

At present, graphite felt or carbon felt is adopted as the typical electrode material attributed to its advantages, including wide operating potentials, high electrical conductivity, good corrosion resistance, and low costs [26,27,28]. However, the original graphite felt shows poor battery performance, primarily due to its hydrophobic property and poor catalytic activity [29]. One promising method to improve hydrophilicity and catalytic activity is the activation of the graphite felt surface [30]. In our work, we have attempted to assess the effect of graphite felt activation with the use of CO_2_ on electrode behavior and catalytic properties of this material in contact with a 4-chlorophenol solution. Graphite felt GFA 10 used in our studies is characterized by good chemical and physical properties according to information supplied by the manufacturer [31,32]. Thus, it can be applied in alkaline batteries in the metallurgy, automobile, and high technologies industries. Taking this into account, we suggest its application also as a catalytic and electrocatalytic material in the degradation of organics present in industrial wastewater. To our best knowledge, this material (GFA 10) has not been applied in wastewater treatment and even has not been characterized as a potential catalytic and electrocatalytic material in the removal of organic pollutants.

Herein, we activated graphite felt GFA 10 in CO_2_ to increase its specific surface area and improve its catalytic and electrocatalytic properties. Subsequently, we determined its morphological characteristics as well as its electrochemical and corrosion characteristics to prove its applicability in the degradation of organic pollutants.

## 2. Results and Discussion

Graphite felt GFA 10 was activated with the application of physical activation in CO_2_ [30,33] for 35 and 70 min and resulted in a change in the GFA surface. The series of graphite felt was denoted as GFA for non-activated material and as GFA-35 or GFA-70 for material activated for 35 and 70 min, respectively. The preparation details of samples are presented in Section 3.1.

### 2.1. Morphological Characterization of GFA

The specific surface area of GFA samples was determined by the Brunauer–Emmett–Teller equation (BET) [34]. The calculations are related to the monolayer volume of adsorbed gas (N_2_) from the isotherm data. GFA graphite felt is not a typical porous material. Due to this fact, the pore volumes were calculated using the Barrett–Joyner–Halenda (BJH) analysis. Parameters of the porous structure of graphite felt samples are summarized in Table 1.

As can be seen, activation in CO_2_ gives an increase in S_BET_ or V about 3–7 times for GFA-35 and GFA-70, respectively, but even these values for GFA-70 are very small in comparison with, e.g., carbon blacks (by an order or two orders of magnitude) [35] or activated carbons (by two or three orders of magnitude) [36]. GFA graphite felt surface is very resistant to CO_2_ action in high temperatures typical for activated carbon production.

However, the increase in GFA-specific surface area is relatively high in comparison with thermal activation of graphite felt under air at 500 °C for 5 h resulting in only a 1.7-fold increase in S_BET_ [37] and in comparison with thermal activation under air at 400 °C for 6 h giving only 1.4-fold increase in S_BET_ [38]. A higher increase in S_BET_ (9.8-fold) was observed during the activation of graphite felt in CO_2_ at 1000 °C for 30 min [30], but the authors did not supply any information on the mass loss caused by burn-off. In the above-mentioned papers, graphite felts were activated in different methods and, under different conditions, were applied in vanadium redox flow batteries. 

**Figure 1:**
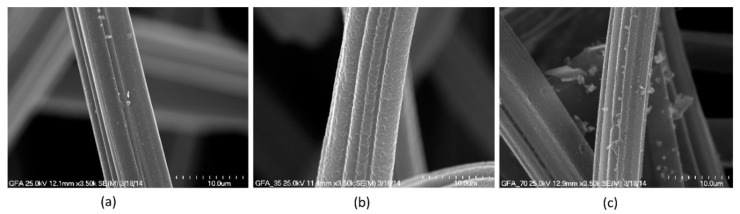
SEM images recorded for GFA (**a**), GFA-35 (**b**), and GFA-70 (**c**) materials.

Individual fibers of GFA before and after activation are shown in SEM images (Figure 1).

**Figure 2:**
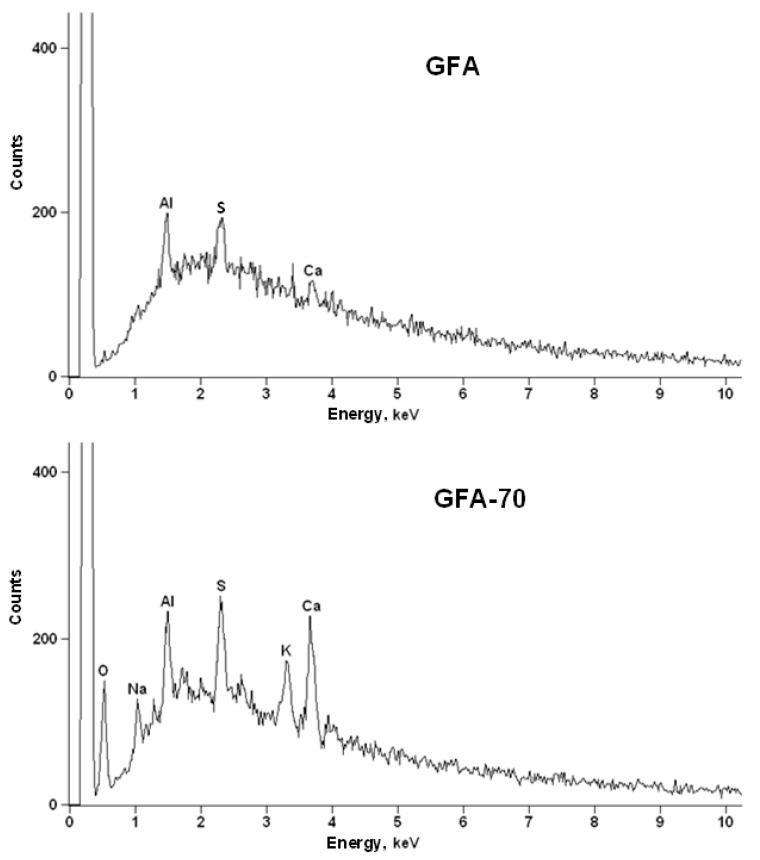
EDS spectra recorded for GFA and GFA-70 materials.

The chemical composition of GFA samples was also investigated using the SEM-EDS method. All samples are composed mainly of carbon. EDS spectra recorded for GFA and GFA-35 were comparable and showed a small number of impurities, i.e., not higher than 1.5%. The longer activation of GFA in CO_2_ (70 min) resulted in a relatively higher amount of impurities. This can be related to the Boudouard reaction occurring on the GFA surface during the activation and leading to a decrease in C content [39,40]. Moreover, the presence of O, Na, and K was observed only in the case of GFA-70 (Figure 2).

The content of the above-mentioned impurities can be changed during the activation of GFA in CO_2_. Probably, in the case of GFA-70, lowering the relative content of carbon and increasing the relative content of O caused these impurities to become visible, and their presence was recognized by the scanning electron microscope. In the case of GFA-35, the content of impurities was intermediate between that observed for GFA and GFA-70 samples. Al is an impurity that can arise from an aluminum table applied in scanning electron microscopes.

### 2.2. Electrochemical Characterization of GFA

Given the potential for wide applications of GFA, especially in the field of electrochemistry, it was necessary to determine its electrochemical characteristics. Furthermore, the effect of GFA activation on its electrochemical properties has to be investigated.

#### 2.2.1. Study of GFA in [Fe(CN)_6_]^4−^/[Fe(CN)_6_]^3−^ System

**Figure 3:**
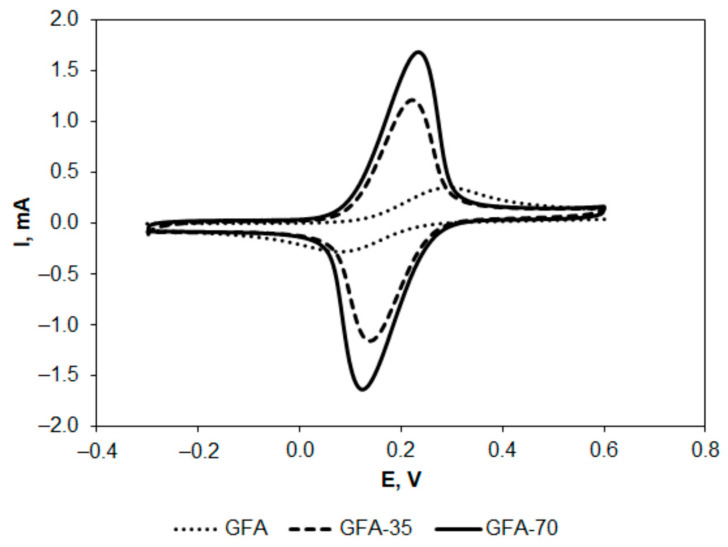
Cyclic voltammograms recorded at GFA electrodes in K_4_[Fe(CN)_6_] (5 × 10^−3^ mol L^−1^ in 0.1 mol L^−1^ KCl) at the scan rate of 5 mV s^−1^.

[Fe(CN)_6_]^4−^/[Fe(CN)_6_]^3−^ system is commonly applied in the determination of electrochemical characteristics of various electrode materials, including carbon electrodes [41,42,43,44,45]. Voltammetric curves recorded at GFA, GFA-35, and GFA-70 electrodes at the scan rate of 5 mV s^−1^ are presented in Figure 3.

**Figure 4:**
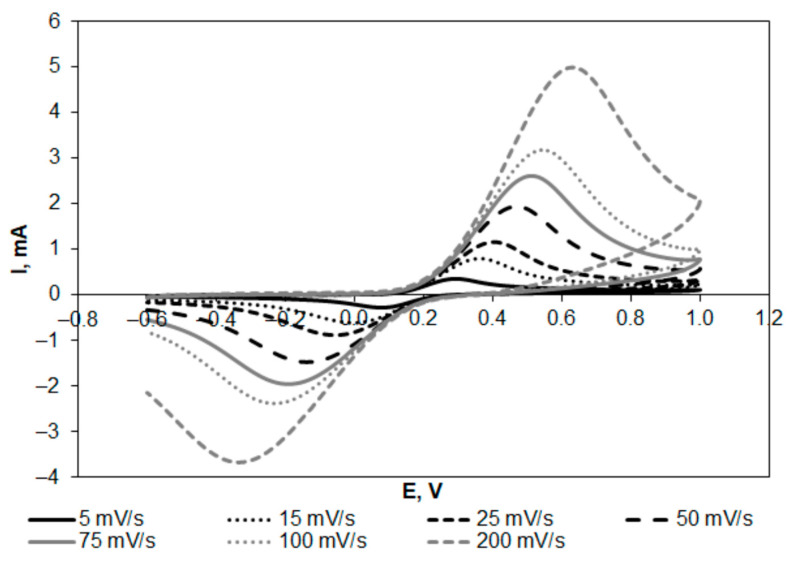
Exemplary cyclic voltammograms recorded at GFA electrodes in K_4_[Fe(CN)_6_] (5 × 10^−3^ mol L^−1^ in 0.1 mol L^−1^ KCl) at the scan rates in the range from 5 to 200 mV s^−1^.

Exemplary voltammetric curves recorded at the GFA electrode at different scan rates in the range from 5 to 200 mV s^−1^ are shown in Figure 4. 

Electrochemical parameters, i.e., peak current, peak potential, a ratio of anodic peak current to cathodic peak current, difference between anodic and cathodic peak potential, and half-wave potential, determined from cyclic voltammetry curves, are listed in Table 2.

The activation of GFA resulted in an increase in the anodic and cathodic peak currents observed in the redox system. In the case of the anodic peak, 35 min activation in CO_2_ caused a threefold increase in the peak current, while 70 min activation resulted in a higher than fourfold increase. Furthermore, the activation resulted in a decrease in the anodic and cathodic peak ratio to almost 1 (Table 2), indicating the reversible nature of the redox couple. The half-wave potential (E_1/2_) calculated using the expression (E_pa_ + E_pc_)/2 was almost constant, showing no effect of the activation. However, the value of ΔEp (the peak-to-peak separation) was clearly lower in the case of activated GFA, indicating more reversible electron transfer in the electrochemical reaction. The increase in anodic and cathodic peak currents also proves that electrochemical oxidation and reduction of the redox couple proceed significantly slower at non-activated GFA. Thus, the electroactive surface area of GFA and the activation effect on its value were determined.

Electroactive surface area (EASA) was calculated from the Randles–Sevcik equation, which can be applied in the case of all electrochemical processes that are controlled by diffusion [46]:

(1) $$I_{p}=2.69\cdot 10^{5}\cdot n^{3/2}\cdot A\cdot D^{1/2}\cdot C\cdot v^{1/2}$$

where *A* is the electroactive surface area, *I_p_* is the peak current, *D* is the diffusion coefficient of the analyte, *n* is the number of transferred electrons, *v* is the scan rate, and *C* is the concentration of the redox molecules in a solution.

**Figure 5:**
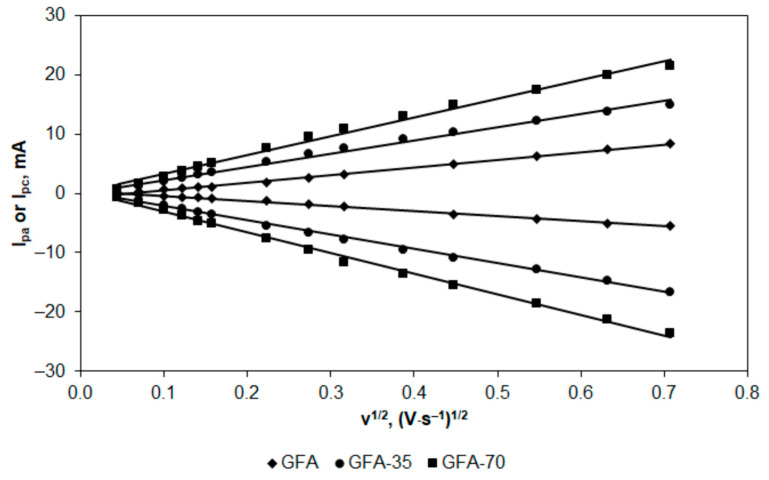
A plot of I_pa_ and I_pc_ against v^1/2^ for GFA, GFA-35, and GFA-70 determined in K_4_[Fe(CN)_6_] (5 × 10^−3^ mol L^−1^ in 0.1 mol L^−1^ KCl) at the scan rates in the range from 5 to 200 mV s^−1^.

Therefore, it was first necessary to confirm the diffusion control in the redox couple system by determining the dependence of I_p_ on the square root of the scan rate, which should be linear. These dependencies are presented in Figure 5.

To additionally confirm the diffusion control for both the anodic and cathodic processes, relationships of log I_p_ vs. log v (v = scan rate) were determined. The equations describing these relationships are shown in Table 3.

The slopes of the I_p_ dependence on v are almost the same for GFA activated in CO_2_ and determined from the anodic and cathodic peaks. Their values slightly exceed the value of 0.5, which is theoretically expected in the case of diffusion control. However, in the case of the non-activated GFA, the slope values are higher (about 0.7), indicating little contribution of adsorption in the reaction control [47,48].

EASA of GFA electrodes was calculated based on the relationships determined for the anodic peak of the redox couple and, for comparison, also based on the relationships obtained for the cathodic peak. The results of calculations carried out taking into account the commonly known values of the diffusion coefficients of the oxidized, and reduced form in the [Fe(CN)_6_]^4-^/[Fe(CN)_6_]^3-^ system equal to 7.63 × 10^−6^ cm^2^ s^−1^ and 6.50 × 10^−6^ cm^2^ s^−1^ [49,50], respectively, are presented in Table 4.

The activation of GFA in CO_2_ significantly affected EASA. GFA-35 activated for 35 min was characterized by EASA twice that of non-activated GFA, while GFA-70 had EASA almost three times that of GFA. Results of EASA determination were comparable for calculations based on the anodic and cathodic peak current except for GFA, which revealed the little contribution of adsorption in the reaction control. Similar relationships were observed for the roughness factor, which is defined as the ratio of EASA to the geometric area of the electrode [51]. Its value determined for GFA-35 from the anodic and cathodic peak current was 2 and 3 times higher, respectively, in comparison with GFA. Higher time of GFA activation in CO_2_ resulted in almost 3 and 4.5 times higher ρ values determined from the anodic and cathodic peak currents, respectively.

**Figure 6:**
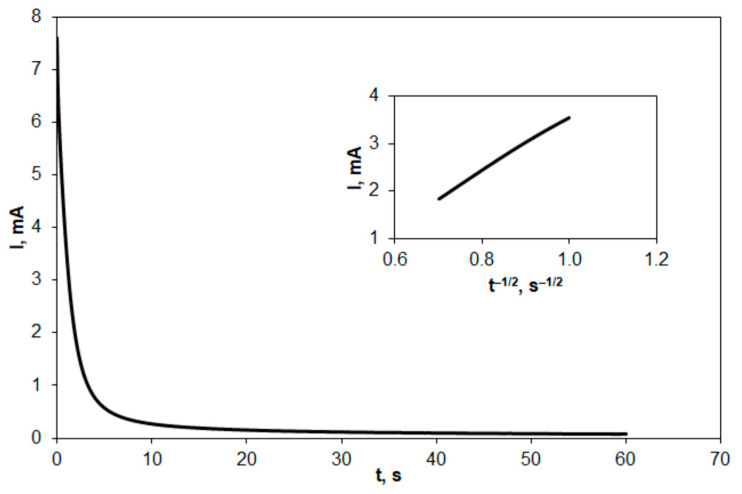
Exemplary chronoamperogram recorded at GFA in K_4_[Fe(CN)_6_] (5 × 10^−3^ mol L^−1^ in 0.1 mol L^−1^ KCl) at the potential of 0.6 V vs. SCE. Inset: Cottrell’s plot of I vs. t^–1/2^.

To confirm the results obtained, the EASA of GFA electrodes was also determined using the chronoamperometry method. Chronoamperograms were recorded in ferri- and ferrocyanide solution for the electrooxidation reaction. An example of a chronoamperogram is presented in Figure 6. The EASA values for the tested electrodes were calculated from the Cottrell equation [52]:

(2) $$I=\frac{n\cdot F\cdot A\cdot D^{1/2}\cdot C}{\pi^{1/2}\cdot t^{1/2}}$$

where *I* is the current intensity, and other parameters have their usual meanings. The results of the calculations are presented together with the roughness factor values in Table 5.

The values of EASA determined from chronoamperograms are clearly higher than those determined from cyclic voltammograms. However, these results confirm that the activation of GFA in CO_2_ increases its EASA by two and three times for activation duration of 35 and 70 min, respectively.

Similarly, the roughness factor also increased two and three times for GFA-35 and GFA-70, respectively.

#### 2.2.2. Electrochemical Behavior of 4-Chlorophenol (4-CP) at GFA Electrode

**Figure 7:**
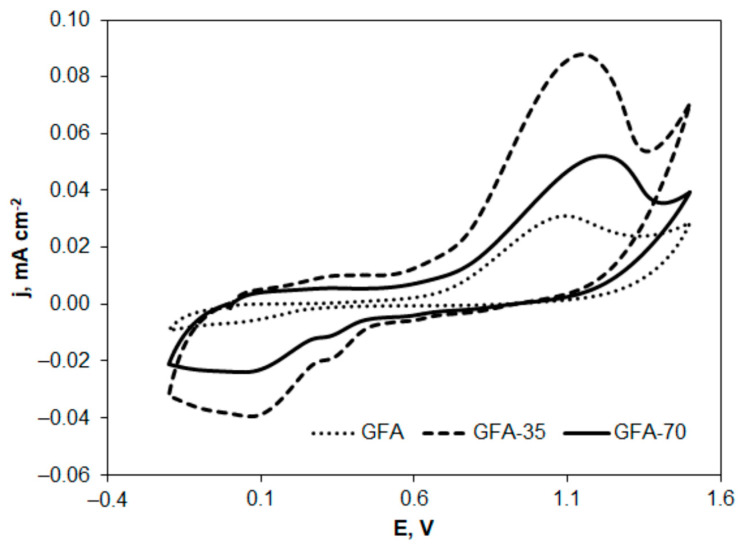
Cyclic voltammograms of 4-chlorophenol solution (1 × 10^−3^ mol L^−1^ in 0.05 mol L^−1^ Na_2_SO_4_) electrooxidation recorded at GFA electrodes; v = 20 mV s^−1^.

**Figure 8:**
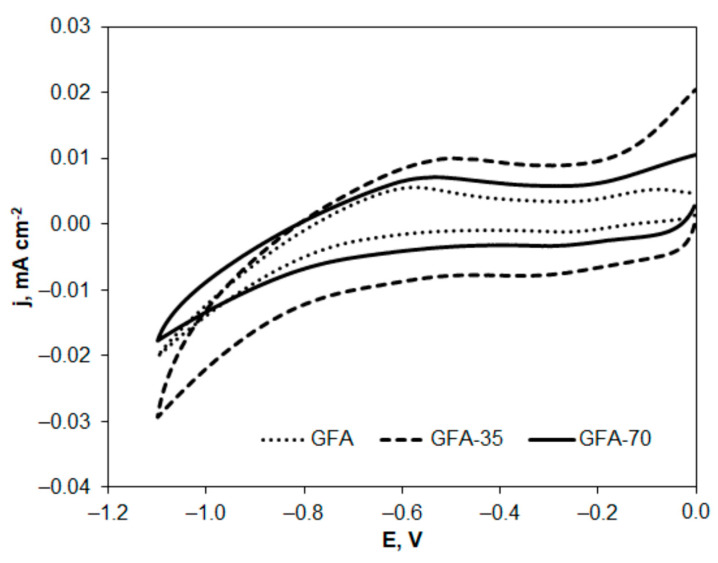
Cyclic voltammograms of 4-chlorophenol solution (1 × 10^−3^ mol L^−1^ in 0.05 mol L^−1^ Na_2_SO_4_) electroreduction recorded at GFA electrodes; v = 20 mV s^−1^.

GFA electrodes can be potentially applied as electrode material in the treatment of industrial wastewater containing 4-CP. Therefore, the electrochemical behavior of 4-CP on GFA electrodes was investigated by cyclic voltammetry. A comparison of cyclic voltammograms recorded at the tested GFA electrodes for electrooxidation and electroreduction of 4-CP is presented in Figure 7 and Figure 8.

The cyclic voltammograms (Figure 7 and Figure 8) show the changes in oxidation or reduction current density versus potential, which were calculated taking into account the previously determined EASA for the electrodes tested. Table 6 presents the comparison of parameters characterizing GFA materials and used in calculations of current densities.

The electrooxidation of 4-CP proceeds in at least one electrode step before the potential reaches the value at which oxygen evolution starts. The activation of GFA in CO_2_ resulted in a higher peak potential of 4-CP oxidation by 57 and 132 mV (Table 7) in the case of GFA-35 and GFA-70, respectively, compared to GFA. This means that a longer activation time of GFA makes the oxidation of 4-CP more difficult at GFA. Furthermore, GFA activation caused a significant increase in the oxidation current of 4-CP. The peak current density determined for GFA-35 was 2.5 times higher compared to the non-activated GFA (Table 7). However, in the case of GFA-70, the longer activation time in CO_2_ resulted in a decrease in the peak current density value, which was still 50% higher compared to the non-activated GFA. The nature of the observed oxidation peaks of 4-CP may indicate a significant effect of the adsorption of this compound on the electrode surface. This was probably the reason for the reduced oxidation peak current of 4-CP on GFA-70 compared to GFA-35. In addition, a longer activation time may have resulted in a deterioration of electrode wettability and an increase in its electrical resistance.

In addition, the electroreduction of 4-CP on the GFA was investigated. Although higher 4-CP reduction currents were observed on GFA-35 and GFA-70, no reduction peaks were developed on the voltamperograms in the potential range up to the potential value at which hydrogen evolution starts.

The results obtained indicate that GFA activated in CO_2_ can potentially be used as electrode material in the electrochemical treatment of 4-CP by electrooxidation. Electrolyses performed at anode potentials higher than the oxygen evolution potential should result in the complete degradation of 4-CP to simple inorganic compounds or to simple organic compounds that are readily biodegradable. Such a process, called electrochemical incineration, requires significant energy consumption but allows the complete degradation of organic compounds present in industrial wastewater. The use of GFAs with a highly developed surface area should make it possible to reduce the electrical energy consumption that often determines the use of the method on a larger scale.

### 2.3. Corrosion Characterization of GFA

The stability of electrode materials, applied as anodes and cathodes in the electrochemical oxidation of organic pollutants present in industrial wastewater, is very important. Corrosion is defined as not only a dangerous process but also an extremely costly problem that affects more than just metals and their alloys. GFA electrodes immersed in wastewater may corrode, especially in the presence of O_2_ and reactive oxygen species (ROS) formed during the electrooxidation of organics. These oxidants react with carbonaceous materials surfaces resulting in electrochemical carbon corrosion, which is thermodynamically favorable at potentials higher than 0.207 V vs. SCE (standard potential of carbon oxidation) [53]. Therefore, it was important to investigate the effect of GFA activation on its corrosion resistance.

**Figure 9:**
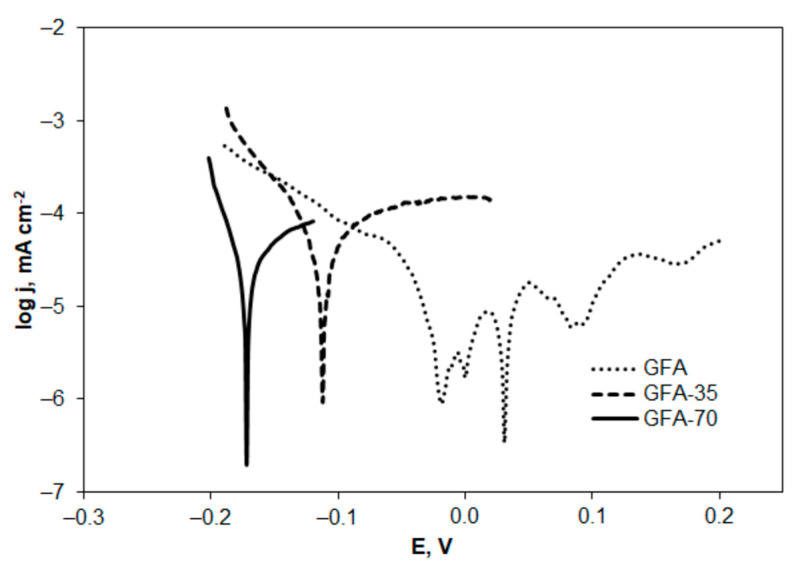
Polarization curves recorded for the tested electrodes in 0.05 mol L^−1^ Na_2_SO_4_.

Assessment of GFA corrosion resistance was performed in a solution of the supporting electrolyte (0.05 mol L^−1^ Na_2_SO_4_) using potentiodynamic polarization sweep preceded by OCP (open circuit potential) determination. The polarization curves were recorded in the potential range of OCP ± 200 mV with the scan rate of 2 mV s^−1^. Examples of the polarization curves recorded at GFA electrodes are shown in Figure 9.

The measured corrosion currents for GFA electrodes, presented in Figure 9, were normalized with respect to EASA values determined for the tested electrodes and shown in Table 6. The electrochemical parameters: anodic and cathodic Tafel slopes (b_a_ and b_c_), corrosion current density (i_corr_), and corrosion potential (E_corr_) determined from the polarization curves are listed in Table 8.

The activation of GFA in CO_2_ resulted in a significant decrease in E_corr_ value. Given that corrosion potential is a thermodynamic parameter that determines susceptibility to corrosion, it can be concluded that GFA-35 and GFA-70 corrode much more easily than non-modified GFA. The longer the activation time, the less corrosion-resistant GFA is obtained. 

Polarization resistance (R_p_) is another corrosion parameter applied in comparison to material corrosion resistance under specified conditions. Activated GFA electrodes are characterized by significantly lower R_p_ values—by 11 and 16 times for GFA-35 and GFA-70 (Table 8), respectively. A lower R_p_ value implies lower corrosion resistance.

A comparison of Tafel slopes (b_a_ and b_c_) indicates that there is probably a change in the mechanism of an anodic reaction during corrosion of activated GFA electrodes. Whereas the cathodic reaction during corrosion probably follows the same mechanism regardless of whether the GFA was activated or not.

The density of corrosion current is a kinetic parameter used in estimating corrosion rates. The highest i_corr_ value was observed in the case of GFA-35 and was about four times higher in comparison with the non-activated GFA. However, increasing the activation time resulted in a reduction of the corrosion current by about 1.8 times in the case of GFA-70 compared to GFA-35. Nevertheless, the corrosion current determined for GFA-70 was still more than two times higher compared to the non-modified GFA, indicating a higher corrosion rate. Given the potential use of activated GFA as electrode material in the electrochemical disposal of organic compounds found in industrial wastewater, the increased durability and corrosion resistance of this material needs to be refined.

### 2.4. Degradation of 4-Chlorophenol with H_2_O_2_

**Figure 10:**
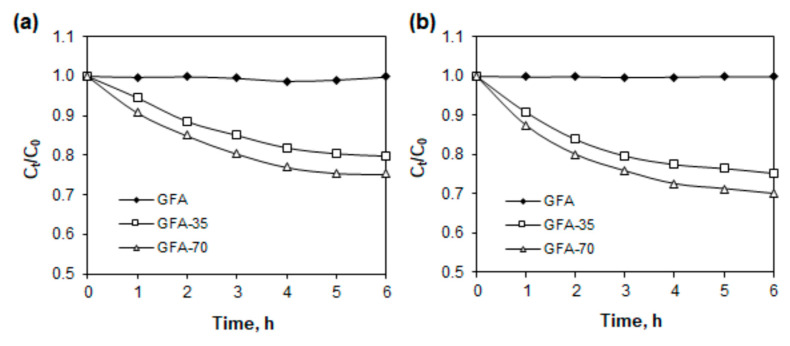
Oxidation of 4-chlorophenol with hydrogen peroxide in the presence of (a) GFA materials from water, (b) 0.05 mol L^−1^ sodium sulfate solution.

Activated carbon and other carbon materials are the most widely used adsorbents for the removal of chlorophenols from water. Carbon materials have also been used in heterogeneous catalysis because they can be used as direct catalysts or as catalyst support for specific needs [54,55]. All materials used in this work were characterized by low BET surface area, and therefore, they cannot be used as adsorbents. However, their usefulness as potential catalysts was investigated. Degradation of 4-chlorophenol from water and 0.05 mol L^−1^ sodium sulfate solutions by hydrogen peroxide in the presence of all three carbon materials is shown in Figure 10.

The percentage loss of 4-CP after 6 h from water without hydrogen peroxide (adsorption) and solutions containing H_2_O_2_ is shown in Table 9.

The results showed that non-activated graphite material (GFA) cannot be used as catalysts. On the other hand, as can be seen in Figure 10 and Table 9, in an aqueous solution in the presence of the GFA-35 and GFA-70 materials was oxidized about 20 and 25% of 4-CP, respectively. From the electrolyte solution was removed about 25 and 30% of 4-chlorophenol, respectively. This fact suggests that the modified graphite materials have catalytic properties. They generate hydroxyl radicals resulting in oxidation of the 4-chlorophenol. The catalytic properties of the graphite materials increase with increasing surface roughness.

The results shown in Table 1, Table 4, Table 5 and Table 9 indicate that increasing the activation time of GFA in CO_2_ to 70 min is favorable, and its further increase above 70 min should allow higher degradation efficiencies of 4-CP. However, it should be considered that a further increase in activation time will result in an increase in mass loss of GFA (Table 10). At some point, the increase in specific surface area and EASA of GFA caused by increasing the activation time will not compensate for the significant mass loss caused by burn-off at 900 °C.

## 3. Materials and Methods

### 3.1. Materials

The commercial graphite felt GFA 10 obtained from SGL Group The Carbon Company (Wiesbaden, Germany) was applied in experiments. Before pretreatment, its specific surface area was below 1 m^2^ g^−1^, according to the manufacturer’s note.

The samples of GFA were heated at the rate of 10 °C min^−1^ to the temperature of 900 °C and then thermally stabilized at this temperature for one hour in a nitrogen stream; then, CO_2_ was introduced, and after the prescribed activation time (35 or 70 min) at 900 °C, it was again replaced with N_2_, and the system was cooled in its stream to the room temperature. Parameters and effects of the activation process are given in Table 10.

### 3.2. Material Characterization

The specific surface area of GFA samples was calculated based on nitrogen adsorption/desorption isotherms measured at 77 K using a Micromeritics ASAP 2020 (Norcross, GA, USA) surface analyzer. The differences in morphology as well as in surface chemistry of GFA samples were determined by scanning electron microscopy SEM (S-4700, Hitachi, Tokyo, Japan) coupled with energy dispersive X-ray analysis EDS (Noran System, Thermo Fisher Scientific, Waltham, MA, USA).

### 3.3. Electrochemical Measurements

All electrochemical measurements were carried out in the three-electrode cell, which was connected to the electrochemical workstation, μATOLAB III (Metrohm Autolab B.V., Utrecht, The Netherlands). NOVA software ver. 2.1 (Metrohm Autolab B.V., Utrecht, the Netherlands)was applied in the analysis of recorded chronoamperograms and voltammograms and in the determination of corrosion parameters. A saturated calomel electrode (SCE) and platinum electrode were used as the reference and counter electrode, respectively. GFA samples with a geometric area of about 2 cm^2^ were applied as the working electrode. Before measurements, all liquid samples were purged with pure argon to remove dissolved oxygen. During measurements, an argon blanket was kept over the solution surface.

Determination of electrochemically active surface area (EASA) of GFA material was performed with the cyclic voltammetry method in K_4_[Fe(CN)_6_] solution (5 × 10^−3^ mol L^−1^ in 0.1 mol L^−1^ KCl) by recording voltammograms at the scan rates in the range from 5 to 200 mV s^−1^. Chronoamperometry was the second method applied in the determination of EASA to confirm the results obtained by the cyclic voltammetry method. Chronoamperograms were recorded in the same solution at the potential of 0.6 V vs. SCE.

The corrosion resistance of GFA material was determined in the supporting electrolyte Na_2_SO_4_ (0.05 mol L^−1^) and was evaluated using the electrochemical technique, measurement of open circuit potential (OCP), followed by potentiodynamic polarization sweep. After the GFA electrode was immersed in the supporting electrolyte, its potential was measured as a function of time. The OCP value was measured for 1 h or less if the OCP value was constant, i.e., the condition dE/dt ≤ 1 μV s^−1^ was fulfilled. The GFA electrodes were cathodically and anodically polarized in the potential range of OCP ± 200 mV with a scan rate of 2 mV s^−1^.

The electrochemical behavior of 4-chlorophenol (4-CP) at GFA material before and after activation in CO_2_ was determined using the cyclic voltammetry method. Voltammograms were recorded at ambient temperature in 4-CP at the concentration of 1 × 10^−3^ mol L^−1^ dissolved in 0.05 mol L^−1^ Na_2_SO_4_. Sodium sulfate was used as a supporting electrolyte.

The 4-chlorophenol (≥99%) was purchased from Sigma-Aldrich (St Louis, MO, USA). All other chemicals used in the experiments were of the analytical reagent grade and were received from Avantor Performance Materials (Gliwice, Poland). The volume of the liquid sample used in voltammetric measurements was 20 mL.

### 3.4. Degradation of 4-CP in the Presence of H_2_O_2_

Batch experiments were performed in Erlenmeyer flasks containing 50 mL of 0.5 mmol L^−1^ solutions of 4-chlorophenol (4-CP) in water or 0.05 mol L^−1^ sodium sulfate. In all tests, the applied concentration of hydrogen peroxide was 5 mmol L^−1^ (ten-fold excess in relation to the 4-CP), and the carbon materials amount was 0.05 g. Erlenmeyer flasks were shaken for 6 h (200 rpm). The concentration of 4-chlorophenol in the solutions was measured by high-performance liquid chromatography with a diode array detector (Shimadzu LC-20, Kyoto, Japan). The separation of analytes was performed using a Phenomenex Luna C18 (4.6 × 150 mm, 3 µm) column (Torrance, CA, USA). The chromatographic conditions were as follows: mobile phase consisted of acetonitrile/water adjusted to pH 3.0 with acetic acid (50/50, *v/v*); flow rate of 0.25 mL/min; analytical wavelengths of 274 nm, which correspond to the maximum absorption peak of the 4-CP. Analytical wavelength was selected based on diode-array spectra taken in real-time analysis. 

## 4. Conclusions

Graphite felt (GFA) appeared to be very resistant to activation in CO_2_ in high temperatures taking into consideration an increase in its specific surface area. Although the S_BET_ of GFA-70 increased seven times in comparison with non-activated GFA, its specific surface area was still relatively low in comparison with carbon blacks or activated carbons. On the other hand, activation of GFA resulted in an increase in electrochemically active surface area, which was two and three times higher for GFA-35 and GFA-70, respectively, in comparison with GFA.

Voltammetric characterization of GFA material in electrochemical oxidation and reduction of 4-chlorophenol indicates that GFA activated in CO_2_ can be potentially applied as electrode material in 4-CP degradation by electrochemical oxidation.

The corrosion resistance of GFA decreased with increasing activation time in CO_2_. GFA-70 corroded at a higher rate in comparison with non-activated GFA. In addition, the corrosion potential of GFA-70 was lower than that of GFA, indicating higher susceptibility to corrosion.

The results of the investigations also indicate that GFA activated in CO_2_ can be applied as a catalytic material in the removal of 4-CP from aqueous solutions, contrary to the non-activated GFA. GFA-35 and GFA-70 generate hydroxyl radicals resulting in oxidation of 4-CP.

Given the potential use of activated GFA as an electrode material or catalytic material in the disposal of organic compounds found in industrial wastewater, the increased durability and corrosion resistance of this material needs to be refined.

## Figures and Tables

**Table 1:** Characteristics of the porous structure of graphite felt before thermal treatment in CO_2_ atmosphere.

Porosity Parameter	GFA	GFA-35	GFA-70
S_BET_, m^2^ g^−1^	0.6	2.1	4.3
V, cm^3^ g^−1^ (from BJH eq)	0.002	0.009	0.017
w, nm (from 4V/S)	10.3	11.2	10.0

S_BET_: specific surface area determined with BET method; V: pore volume; w: pore diameter.

**Table 2:** CV parameters determined from cyclic voltammograms recorded in K_4_[Fe(CN)_6_] (5 × 10^−3^ mol L^−1^ in 0.1 mol L^−1^ KCl) at the scan rate of 5 mV s^−1^.

GFA Material	I_pa_, mA	E_pa_, V	I_pc_, mA	E_pc_, V	I_pa_/I_pc_	ΔE_p_, V	E_1/2_, V
GFA GFA-35 GFA-70	0.350 1.190 1.659	0.294 0.221 0.234	−0.283 −1.163 −1.656	0.079 0.139 0.124	1.24 1.02 1.00	0.215 0.082 0.110	0.187 0.180 0.179

I_pa_ and I_pc_: anodic and cathodic peak current; E_pa_ and E_pc_: anodic and cathodic peak potential; I_pa_/I_pc_: a ratio of anodic peak current to cathodic peak current; ΔE_p_: difference between anodic and cathodic peak potential; E_1/2_: half-wave potential.

**Table 3:** Dependences of log I_p_ vs. log v determined for the tested GFA materials in K_4_[Fe(CN)_6_] (5 × 10^−3^ mol L^−1^ in 0.1 mol L^−1^ KCl).

GFA Material	Equation	R^2^
Anodic Peak		
GFA GFA-35 GFA-70	y = 0.726x − 1.777 y = 0.577x − 1.544 y = 0.583x − 1.383	0.9996 0.9917 0.9916
Cathodic Peak		
GFA GFA-35 GFA-70	y = 0.713x − 1.935 y = 0.594x − 1.517 y = 0.596x − 1.357	0.9911 0.9931 0.9914

**Table 4:** Electroactive surface area (EASA) and roughness factor (ρ) of the tested GFA materials calculated from the dependence of I_pa_ and I_pc_ vs. v^1/2^ determined in K_4_[Fe(CN)_6_] (5 × 10^−3^ mol L^−1^ in 0.1 mol L^−1^ KCl).

GFA Material	Anodic Peak			Cathodic Peak		
	I_pa_ vs. v^1/2^ Slope	EASA, cm^2^	Roughness Factor (ρ)	I_pc_ vs. v^1/2^ Slope	EASA, cm^2^	Roughness Factor (ρ)
GFA GFA-35 GFA-70	1.238 × 10^−2^ 2.482 × 10^−2^ 3.566 × 10^−2^	18.1 36.2 52.0	8.6 17.2 24.8	−8.605 × 10^−3^ −2.594 × 10^−2^ −3.728 × 10^−2^	11.6 34.9 50.2	5.5 16.6 25.1

**Table 5:** Electroactive surface area (EASA) and roughness factor (ρ) calculated for the tested GFA materials from chronoamperograms recorded at the tested materials in K_4_[Fe(CN)_6_] (5 × 10^−3^ mol L^−1^ in 0.1 mol L^−1^ KCl) at the potential of 0.6 V vs. SCE.

GFA Material	I vs. t^−1/2^ slope	EASA, cm^2^	Roughness Factor (ρ)
GFA GFA-35 GFA-70	5.878 × 10^−3^ 1.110 × 10^−2^ 1.752 × 10^−2^	42.4 80.0 126.2	20.2 38.1 60.1

**Table 6:** Parameters characterizing GFA materials applied in recording cyclic voltammograms in the solution of 1 mM 4-chlorophenol (0.05 M Na_2_SO_4_) and presented in Figure 7 and Figure 8.

GFA Material	EASA, cm^2^ g^−1^	EASA, cm^2^ (Electrode)
GFA	920	18.0
GFA-35	1840	36.1
GFA-70	3045	51.6

**Table 7:** Comparison of E_p_ and i_p_ values for electrooxidation of 4-CP on GFA electrodes.

GFA Electrode	E_p_, V	i_p_, mA cm^−2^
GFA	1.095	0.031
GFA-35	1.152	0.078
GFA-70	1.218	0.047

**Table 8:** Corrosion parameters of GFA materials determined in 0.05 mol L^−1^ Na_2_SO_4_.

GFA Material	E_corr_, V	i_corr_, mA cm^−2^	R_p_, kΩ	b_a_, mV dec^−1^	b_c_, mV dec^−1^
GFA GFA-35 GFA-70	0.031 −0.112 −0.172	8.00 × 10^−6^ 3.28 × 10^−5^ 1.78 × 10^−5^	102.840 9.225 6.421	125.2 38.8 23.7	47.1 72.1 47.1

**Table 9:** The percentage removal of 4-chlorophenol after 6 h.

Solution of 4-CP, Composition	GFA Material		
	GFA	GFA-35	GFA-70
Water without H_2_O_2_	0%	0%	1.0%
Water + H_2_O_2_	0.2%	20.2%	24.7%
0.05 mol L^−1^ Na_2_SO_4_ + H_2_O_2_	0.2%	24.9%	30.1%

**Table 10:** Parameters of activation process with CO_2_ in laboratory vertical oven at 900 °C. The heating rate to activation temperature was 10 °C min^−1^. The time of stabilization before the activation process was 1 h (in a nitrogen stream of 30 dm^3^ h^−1^).

Parameters of Activation Process	Time of Activation, min	Initial Sample Mass, g	Mass of Activated GFA, g	Mass Loss, %
900 °C, CO_2_, 30 dm^3^ h^−1^	35	14.8	12.8	13.5
900 °C, CO_2_, 30 dm^3^ h^−1^	70	14.4	9.2	36.4

GFA samples activated for 35 and 70 min were labeled GFA-35 and GFA-75, respectively.

## Data Availability

Not applicable.

## Sample Availability

Samples of the compounds in small amounts are available from the authors.
